# The ten test for facial sensory assessment: A scoping review

**DOI:** 10.1016/j.jpra.2026.08.023

**Published:** 2026-08-20

**Authors:** Adi M. Friedman, Gregory H. Borschel

**Affiliations:** a Indiana University School of Medicine, Indianapolis, IN, USA; b Indiana University Health, Indianapolis, IN, USA

**Keywords:** Ten test, Facial sensation, Sensory assessment, Sensory nerve injury, Nerve injury, Nerve repair, Nerve recovery, Nerve graft, Trigeminal nerve, Trigeminal reconstruction, Cranial vault reconstruction, Facial transplantation, corneal neurotization

## Abstract

Practical and reliable assessment of facial sensation is critical in plastic and reconstructive surgery given its role in mastication, speech, and communication. The Ten Test (TT), a sensory assessment based on comparison between normal and affected sensation, emerged as an alternative to calibrated monofilaments and has been validated extensively in the upper extremity. The goal of this scoping review was to map the existing evidence regarding TT assessment of facial sensation and identify gaps that warrant further investigation. Seven relevant publications were identified, comprising five clinical studies and two descriptive sources. The TT was applied across a range of trigeminal nerve injuries including facial transplantation, iatrogenic nerve injury, and craniosynostosis to assess postoperative sensory recovery. Use extended to intraoral and intranasal mucosa in select contexts, and the TT also played a role in preoperative evaluation of corneal neurotization candidates. This review summarizes the findings and their clinical relevance, including barriers to validation and limitations of evidence, to help improve perioperative facial sensory assessment.

## Introduction

Assessment of facial sensation is critical in plastic and reconstructive surgery given its role in mastication, speech, breathing, and communication. The primary sensory nerves of the face include the trigeminal nerve (CNV), which provides sensation to the face, tongue, teeth, oral mucosa, nasal mucosa, and cornea,^1^ and the great auricular nerve, which innervates the mandible, auricle, and earlobe.^2^ Injury to these nerves can result in sensory disturbances, intra-oral or nasal mucosal injuries, altered taste sensation, neurotrophic keratopathy, dysphonia, or dysphagia.^3^, ^4^, ^5^, ^6^ These complications can significantly impair functionality and quality of life. Given the serious nature of such deficits, it is imperative that plastic surgeons have precise, reliable, and practical means of evaluating facial sensation in their patients.

Clinical sensory tests assess facial sensation by targeting different nerve receptors in the skin through pressure thresholds and spatial discrimination. Slowly adapting receptors sense sustained touch while rapidly adapting receptors detect movement across the skin.^7^ Free nerve endings contribute to temperature and pain sensation with lower specificity.^7^

There are a variety of tests that target these pathways to assess facial sensation, with two of the most commonly used and best characterized being the Weinstein Enhanced Sensory Test (WEST) and the two-point discrimination test (2PD). The WEST integrates Semmes-Weinstein monofilaments to gauge the pressure threshold, defined as the softest stimulus eliciting a sensory response in an area of skin. While the WEST has demonstrated high reliability, it has several drawbacks that limit its clinical utility. Sensation is estimated on a logarithmic scale with increasingly greater intervals, representing a range rather than a true threshold value.^8^ Furthermore, it is relatively expensive, requires precise calibration, and demands appropriate training.

The 2PD measures innervation density by applying static or moving pressure to two points on the skin and recording the smallest distance at which the two stimuli are perceived as distinct. Although handheld tools such as the Disk-Criminator are readily available, the 2PD has been found to be less sensitive in patients with sensory disturbance,^9^^,^^10^ demonstrates lower inter-rater reliability,^11^ and performs better with younger patient populations.^12^ In clinical practice, the resource-dependent nature of the WEST and the limited precision of the 2PD diminish their utility in pre-operative assessment.

Therefore, there remains a need for a clinical test that assesses facial sensation in a practical, efficient, and reliable way. The Ten Test (TT), proposed by Berish Strauch in 1997, was developed as a reliable alternative to calibrated monofilaments for assessing upper extremity sensation. The TT is based on a ratio between normal and reduced sensation, whereby patients rate sensation in an affected area on a scale of 1–10, with 10 representing sensation in a comparably innervated, unaffected region.^13^

Since its introduction, the TT has been well characterized in the upper extremity demonstrating strong correlation with WEST testing in hand trauma patients (Spearman rank coefficient of -0.71) ,^13^^,^^14^ excellent inter-rater reliability,^15^ high intra-rater reliability (with wider variations in findings) ,^16^ and superior performance than WEST and 2PD in predicting early sensory loss from chronic nerve compression.^16^ Despite these merits, the TT has not been validated for assessing facial sensation to the same extent as the upper extremity. Although inherently subjective, the TT provides a straightforward measure of perceived sensory deficits that avoids the logistical constraints of other commonly used sensory tests. Given that the literature describing TT application to facial sensory assessment has not previously been summarized, a scoping review was conducted to map the available evidence, characterize its clinical application, and identify gaps that may warrant further investigation.

## Methods

This scoping review was conducted to identify the existing evidence regarding the Ten Test (TT) in the assessment of facial sensation. A demonstration of the Ten Test technique for facial sensation is provided in Supplementary Video 1 in a patient with impaired left trigeminal sensation after a tumor resection. All aspects of the review were conducted in accordance with PRISMA-ScR guidelines.

### Search strategy

A comprehensive literature search of major databases including PubMed, Embase, and Google Scholar was conducted through April 2026. The search strategy combined terms related to the “Ten Test” with relevant facial anatomy and sensory keywords. Title and abstract fields were searched in PubMed and Embase. For Google Scholar, the first 200 results sorted by relevance were screened. Reference lists of all included articles were manually reviewed to identify additional eligible studies. The complete search strategies for each database are provided in Supplementary Appendix 1.

### Eligibility criteria

Studies were eligible for inclusion if they reported use of the Ten Test as a clinical tool to measure facial sensation in human subjects. There were no restrictions placed on patient population, underlying diagnosis, or clinical setting. Eligible study designs included randomized trials, cohort studies, case-control studies, case series, and case reports. Relevant clinical textbook chapters and pre-operative workups describing clinical application of the TT were included as well.

Review articles, conference abstracts without full text, animal studies, cadaveric studies, and nonclinical investigations were excluded from the final analysis. However, their reference lists were screened to identify additional eligible sources. Studies were also excluded if the term “ten test” was used in a context unrelated to the clinical sensory test. Only articles published in English were considered.

### Selection and screening

All records identified through the database searches were exported and duplicates were removed. Titles and abstracts were screened for mention of the TT in facial sensory assessment. Potentially eligible articles underwent full-text review. Screening and eligibility assessment were performed by a single reviewer.

Review articles and other secondary sources were excluded from the final analysis if they did not contain original data pertaining to TT facial sensory assessment. Their reference lists were screened to identify additional eligible studies.

### Data extraction and synthesis

The full texts of all included studies were reviewed in detail, and relevant data were extracted using a standardized data collection form. Variables included clinical context of TT application, patient population, anatomical region assessed, comparator sensory tests, and key study findings. Given the heterogeneity of study designs and outcomes, formal statistical analysis was not undertaken. The findings were synthesized to characterize how the TT has been applied in the clinical literature and to identify areas where evidence remains limited. Descriptive statistics were used where appropriate.

## Results

### Study selection

**Fig. 1:**
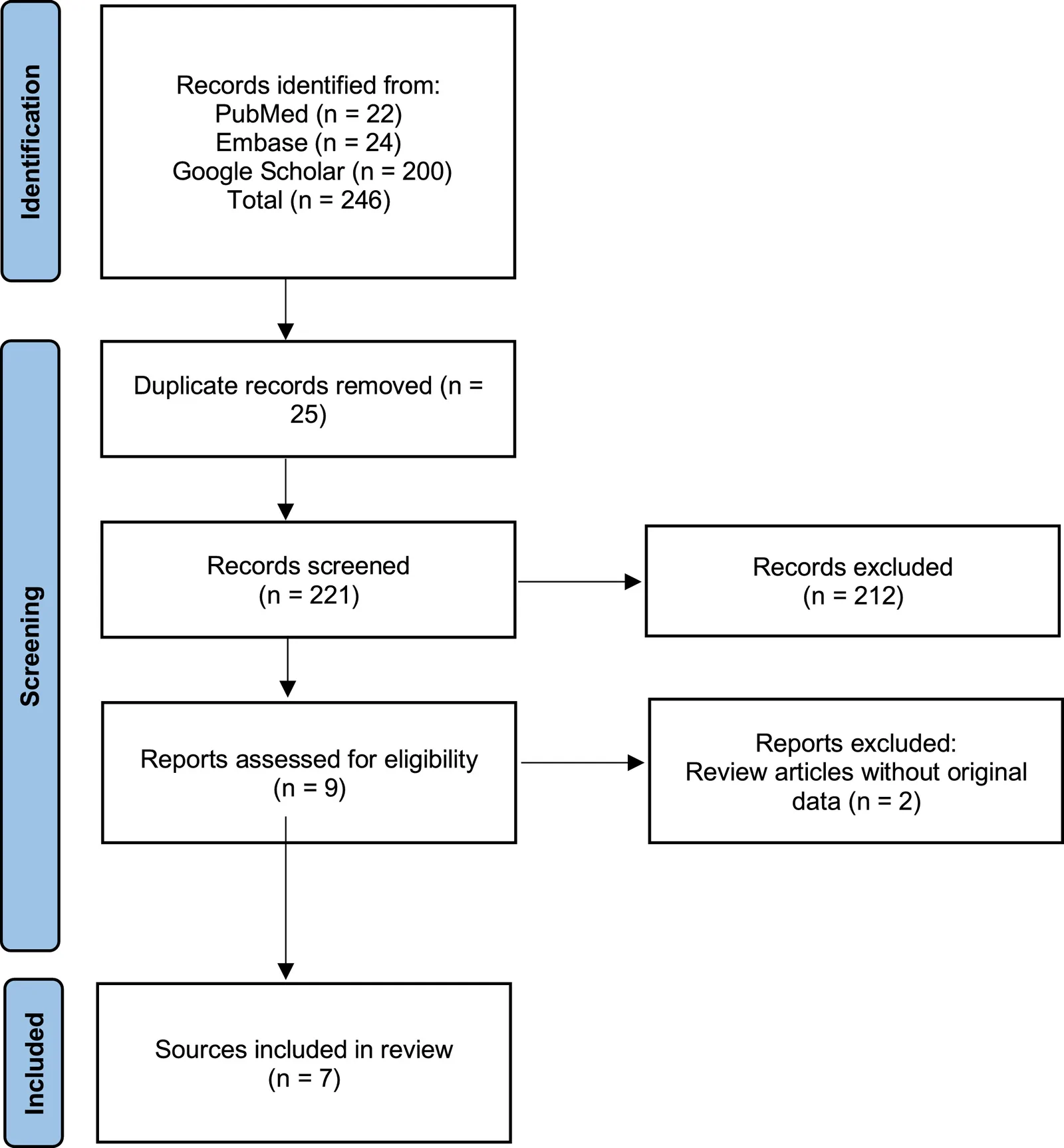
PRISMA flow diagram of study selection. Flow diagram illustrating the identification, screening, eligibility assessment, and inclusion of sources in accordance with PRISMA-ScR guidelines.

A systematic literature search was conducted using PubMed, Embase, and Google Scholar to identify clinical applications of the Ten Test (TT) for facial sensory assessment. The study selection process is summarized in Fig. 1. Search results were exported to EndNote for deduplication and screening. After removal of duplicate search results, 221 records underwent title and abstract screening. A total of nine records met the criteria for full-text review. Of these, two review articles were excluded because they contained overlapping primary sources. Seven sources were included in the review, five of which were clinical studies, while two were descriptive secondary sources.

### Study characteristics

Five clinical studies recording the use of the Ten Test in assessing facial sensation were identified, each measuring sensibility in trigeminal nerve distributions across various contexts. These included cranial vault reconstruction in children, iatrogenic inferior alveolar nerve injury, facial transplantation, corneal neurotization, and cross face nerve grafting. The primary outcome measured by the TT was facial sensory recovery post-operation. In addition, it was used to identify donor trigeminal nerve branches for corneal neurotization. The clinical sources identified in this review represent a mid to low level of evidence, with three studies being retrospective case series, one being a cross-sectional case-control, and one being a case report. Study characteristics are summarized in Table 1.

**Table 1:** Study characteristics.

**Author/Year**	**Institution**	**Study Design**	**Level of Evidence**	**Study Population**	**n**	**Mean Age (years)**	**Follow Up (months)**	**Primary Outcome**
Dengler et al. ,^17^ 2019	University of Toronto	Cross-sectional case-control	III	Children with isolated craniosynostosis	28	9.6	NR	Facial sensibility comparison
Mundinger et al. ,^18^ 2012	Johns Hopkins Hospital, University of Washington, University of Texas Southwestern Medical School	Retrospective case series	IV	Iatrogenic IAN injury	5	49	47.8	Sensation recovery
Wall et al. ,^19^ 2016	Brigham and Women’s Hospital, Harvard Medical School	Retrospective case series	IV	Facial transplant recipients	6	42.2	37.5	Functional and sensory outcomes
Woo et al. ,^20^ 2022	The Hospital for Sick Children, Toronto, Ontario	Retrospective case series	IV	Corneal neurotization patients	23	15.6 ± 13.6	43.38±20.63	Donor nerve assessment
Greenhowe et al. ,^21^ 2026	The Hospital for Sick Children, Toronto, Ontario and Indiana School of Medicine	Retrospective case study	V	Cross face nerve graft	1	9	18	Sensory recovery

IAN - inferior alveolar nerve; NR – not reported.

### Application and findings

Sensory assessment using the TT focused primarily on trigeminal nerve distributions of facial skin but was also applied to intraoral^19^^,^^21^ and intranasal mucosa.^21^ The TT was frequently paired with comparator sensory tests, most commonly the WEST (appeared in three studies), as well as sharp-dull discrimination, light touch, and pinch testing. Across studies the TT was rarely used as a primary outcome measure and instead functioned as a part of a more holistic assessment strategy. In several articles, the TT was applied to anatomical regions less accessible to traditional quantitative sensory testing. Reporting of TT methodology varied across studies, with some providing limited procedural detail. Application of the TT spanned multiple trigeminal nerve branches and a wide age range, from pediatric cohorts to adult populations. A summary of key evidence is provided in Table 2.

**Table 2:** Summary of key evidence.

**Author/Year**	**Region Tested**	**TT Methodology Reported**	**TT Role**	**Comparator Tests**	**Key Findings**	**Key Limitations**
Dengler et al., 2019	SO, ST, ZT, ZF distributions	TT performed; cited Strauch et al.; administration details not reported	Adjunct	WEST; self-report	No significant sensory deficits vs controls	Small sample; cross-sectional design
Mundinger et al., 2012	IAN distribution (mandible)	Self-performed (x3 per patient) according to Strauch et al.	Primary	None	Mean TT score 3.3 at follow up	Small sample; subjective reporting
Wall et al., 2016	Perioral skin; labial mucosa	TT performed; cited Strauch et al.; administration details not reported	Primary	Sharp/dull; light touch; thermal	TT scores 5–9 (perioral); 6–9 (labial)	Small sample; heterogeneous follow-up
Woo et al., 2022	Facial and cervical regions	TT performed; administration details not reported	Adjunct	WEST; pinch test	Used for facial sensory mapping	Small sample; retrospective design; TT adjunct outcome
Greenhowe et al., 2026	Nasal septum; tongue	TT performed; administration details not reported	Primary	WEST	TT 5/10 (septum), 8/10 (tongue) at 18 months	Single case

TT – Ten Test; WEST – Weinstein Enhanced Sensory Test; SO – Supraorbital; ST – Supratrochlear; ZT – Zygomaticotemporal; ZF – Zygomaticofacial; IAN – Inferior alveolar nerve.

TT scores demonstrated a broad range (3.3–9) across multiple trigeminal nerve distributions in the facial and perioral skin, intranasal mucosa, intraoral mucosa, and tongue. This variability reflects heterogeneity within the included literature which differed with respect to anatomical region tested, follow-up duration, comparator modalities, patient populations, and primary study objectives. The overall evidence base remains limited by small sample sizes, predominantly retrospective study designs, and inconsistent reporting of TT methodology.

Two additional publications provided descriptive guidance on the clinical application of the TT without reporting patient specific outcomes and were therefore excluded from the quantitative evidence tables. Yang and Tarufo (2020) proposed the TT as a quick method for comparative facial sensibility assessment prior to secondary nerve reconstruction.^22^ Daeschler et al. (2023) recommended incorporating the TT into preoperative evaluation for corneal neurotization (along with the WEST and pinch test) to assist in identification of potential donor nerve distributions.^23^

### Summary of evidence

Several clinical patterns emerged from the synthesized evidence. The TT was most commonly used to assess postoperative sensory recovery in trigeminal distributions. Although primarily applied to facial skin, its use extended to intraoral and intranasal mucosa. The TT also appeared to play a role in the preoperative evaluation of candidates for corneal neurotization. Across studies the TT was typically incorporated as a part of a multimodal sensory assessment strategy and was applied across a broad age range. However, the current evidence is limited by retrospective study design, small sample size, and heterogeneous reporting of TT methodology.

## Discussion

### Principal findings and knowledge gaps

This scoping review identified limited clinical evidence describing use of the Ten Test (TT) for assessment of facial sensation. The findings demonstrate application across a range of trigeminal nerve injuries including craniosynostosis reconstruction, iatrogenic nerve injury, facial transplantation, and preoperative evaluation for corneal neurotization. Despite these applications, no studies formally evaluate the diagnostic accuracy, reliability, or predictive value of the TT for facial sensory impairment. This contrasts with the upper extremity, for which the TT has been extensively characterized and validated, highlighting a substantial gap in the facial sensory literature. Establishing comparable evidence for facial application represents an unmet need.

### Barriers to validation

Validation of sensory assessment in the face presents unique challenges compared with other anatomical regions. Facial sensation is mediated by complex and regionally variable contributions from both cutaneous and mucosal receptors, with sensory thresholds that vary by modality of discrimination, anatomical location, and patient age.^24^^,^^25^ In addition, common objective comparator tests such as the Weinstein Enhanced Sensory Test (WEST) or two-point discrimination (2PD) focus on specific sensory modalities and may not reflect overall sensibility.

Interpretation of sensory deficits may also be complicated by communication between the trigeminal and facial nerve distribution and variability in clinical presentation.^7^ Furthermore, reliance on the contralateral face as an internal control may be limited in clinical settings involving bilateral sensory impairment, as well as by baseline sensory asymmetries or subclinical variation in many patients. Thus, the TT may be less applicable in craniofacial procedures where an unaffected reference region is not available. These factors, combined with heterogeneous surgical populations and a clinical preference for objective sensory measures likely contribute to the relative scarcity of validation studies for facial application of the TT observed in this review.

### Clinical implications

Despite the challenges associated with formal validation, the TT exhibits several features that may support its clinical utility in facial sensory assessment. Findings from this review highlighted versatility, with application across multiple trigeminal distributions in both preoperative and postoperative settings. Notably, the TT was used in anatomical regions such as the labial mucosa, nasal septum, and tongue, suggesting potential utility in areas less amenable to traditional modalities such as the WEST or 2PD.

In one study, the TT was reported to be consistent with Semmes-Weinstein monofilament assessment in detecting normal sensibility across several trigeminal distributions in pediatric craniosynostosis cohort. While this observation is limited in scope, it suggests that the TT may provide clinically comparable estimates of sensibility in certain contexts. More broadly, the TT offers several practical advantages including low cost, minimal equipment, ease of administration in bedside setting, and lack of required specialized training making it ideal for perioperative surgical assessment.

### Limitations

This review is limited by the overall quality of the available evidence. Most identified studies employed retrospective designs with small sample sizes and heterogeneous reporting of TT application, limiting the ability to draw conclusions regarding its clinical performance. In addition, the TT was frequently included as an adjunct rather than primary outcome measure, further obscuring the interpretation of its utility.

This review is also subject to methodological limitations. Literature search, study screening, and study selection were performed by a single reviewer potentially introducing selection bias and reducing comprehensiveness. Although multiple databases were searched, relevant evidence may have been overlooked due to inconsistent indexing or variability in terminology describing the Ten Test.

## Conclusions

The practical advantages that motivated the development and acceptance of the TT in the hand – namely its low cost, ease of administration, and high reliability – remain relevant in facial sensory assessment. This scoping review found that the TT has already been adopted across a range of trigeminal nerve distributions and surgical settings, often alongside traditional sensory modalities. Although available evidence is limited by small sample sizes and heterogeneous methodology, these findings suggest that further investigation is warranted to clarify the clinical contexts in which the TT provides meaningful facial sensory information. Prospective validation studies are needed to determine its diagnostic accuracy, intra- and inter-rater reliability, and correlation with established sensory modalities across trigeminal distributions. Moreover, standardization of TT application in the face, with consistent scoring and explicit procedure, will help eliminate ambiguity in administration and improve comparability across studies. Such efforts may permit the incorporation of the TT as a practical and effective complement to existing measures of facial sensibility in the surgical setting.

## Funding

None.

## Ethical approval

Not required.

## Declaration of competing interest

None declared.

## Appendix Supplementary materials

Image, application 1

Image, video 2 dummy alt text
