# Supplementary material for: The ten test for facial sensory assessment: A scoping review
Source: JPRAS Open. 2026 Aug 20;52:186–91. doi: 10.1016/j.jpra.2026.08.023 (PMC13564054; doi:10.1016/j.jpra.2026.08.023)
Supplement: Supplementary file 1 [file mmc1.docx]

**Supplementary Appendix 1. Search Strategy**

Pubmed (searched April 2026)

("Ten Test"[tiab] OR "10 Test"[tiab] OR "ten-test"[tiab] OR "Ten-Test"[tiab]) AND

(facial[tiab] OR face[tiab] OR trigeminal[tiab] OR orofacial[tiab] OR lip[tiab] OR cheek[tiab] OR forehead[tiab] OR cornea[tiab] OR "inferior alveolar"[tiab] OR mental nerve[tiab] OR perioral[tiab])

Embase (search April 2026)

('ten test':ti,ab OR '10 test':ti,ab OR 'ten-test':ti,ab) AND

(facial:ti,ab OR face:ti,ab OR trigeminal:ti,ab OR orofacial:ti,ab OR lip:ti,ab OR cheek:ti,ab OR forehead:ti,ab OR cornea:ti,ab OR 'inferior alveolar':ti,ab OR 'mental nerve':ti,ab OR perioral:ti,ab)

Google Scholar (searched April 2026)

"Ten Test" (facial OR trigeminal OR face OR orofacial) AND (sensation)
